# A Transitional Experience: Basic Science to Clinical Rotation

**DOI:** 10.31729/jnma.8077

**Published:** 2023-03-31

**Authors:** Bishal Pradhan, Prerana Singh Rokaha, Ashmina Lama

**Affiliations:** 1 Nepalese Army Institute of Health Sciences, Sanobharyang, Kathmandu, Nepal; 2 Kathmandu Medical College and Teaching Hospital, Sinamangal, Kathmandu, Nepal

**Keywords:** *clinical medicine*, *communication*, *educational activity*, *leadership*

## Abstract

Bachelor of Medicine and Bachelor of Surgery is a roller coaster journey that is educational yet emotional. Because of how the context and responsibilities change throughout time, learning is exciting. Nevertheless, the exposure to basic science during this course teaches us to be diligent, committed, and compassionate, and it also gets us ready for the next stage of clinical practice. For us as students, the primary areas that change as a result of this transformation are professional networking, workload, patient interaction, time management, leadership, and communication skills. In this journey, the transition is inevitable so we need to adapt to change seamlessly. Additionally, extracurricular activities play a significant role in this journey.

## INTRODUCTION

The journey through basic science while studying Bachelor of Medicine and Bachelor of Surgery (MBBS) is an emotional yet introspective experience that is crucial to the development of our professional identities.^1^ Meanwhile, clinical classes encourage medical students in a variety of ways, including their academic performance, clinical competency, communication abilities, and self-assurance.^2^ Due to the ongoing scientific revolution in medicine, it is imperative that MBBS graduates need to have a solid foundation in the basic sciences and through clinical experiences, students can put their learning to use and pick up new abilities under the supervision of a preceptor.^3^ The basics and clinical are two essential components of the MBBS curriculum, and as we progress from the basics to the clinical, there is a change in study subjects, as well as how we feel and what we experience as medical students. When students comprehend not only "what happens," but also "why it happens," and "why they have to study," less grey areas and a better picture of black and white emerge as it seemed to us when transitioning from basics to clinical.^4^

### 1. Our areas of study

Medical aspirants enter medical school with high expectations for the novel knowledge they would acquire in the medical sector, yet the first two years of study are only addressed in brand-new books and lectures. We study the fundamentals of the organ-system curriculum and gain knowledge of the identification and description of human body structures in anatomy, the functions and mechanisms of human systems in physiology, the study of chemical processes occurring within and relating to humans in biochemistry, the causes and effects of disease or injury in pathology, the biology of microscopic organisms that cause deviations from the normal state of the body in microbiology, and the interactions of drugs with the system in pharmacology.^5^ Community medicine which focuses on preventing disease, understanding the causes and course of disease in populations, and understanding how the environment and society affect health and disease is also included in this curriculum. The practicals that support the ideas are also presented side by side with the theories to aid in learning.

Either basic science or clinical rotation, one thing that tags along are the pounding subjects, fresh but still a lot. However, it's astonishing since practising physicians and surgeons deliver the lectures and finally, the students have the opportunity to engage with patients and gain clinical experience. The new subjects that are introduced in the clinical years are Internal medicine, which deals with the prevention, diagnosis, and treatment of tropical diseases; paediatrics, which aids in the medical care of infants, children, adolescents, and young adults; surgery, which deals with the manual and instrumental treatment of injuries, diseases, and other disorders; and gynaecology and obstetrics, which focuses on the care of women during pregnancy and childbirth as well as the diagnosis and treatment of diseases of the female reproductive system. Since the corresponding subjects are covered in lectures as well as at the patient's bedside, the learning-by-doing approach is employed during the clinical years.

### 2. Our experience

The majority of young people in Nepal choose to enrol in the MBBS program, which is highly esteemed by parents and society but this journey needs resilience and adaptability. While recollecting the memories of our initial days in medical school, we were all anxious, eager, and oblivious because everything was new, including friends, teachers, and course materials. At first, medical terminologies are confusing and overwhelming and fleets over head, practical classes feel unfamiliar and challenging. Everything about the first anticipated trip to the dissecting room—the skulls, the formalin scent, the first glimpse of the cadaver, and the first use of scalpel and forceps to open those cadavers and dissect them — is exhilarating, though a little terrifying. The first time we attempted to make a blood smear from our own blood was one of the earliest and most exciting basics experience. Wearing a white coat is electrifying, it makes us feel like doctors for the first time though we are merely fledglings in the medical field. Things can get chaotic and confusing at times. However, medical students should remain motivated and always learn from mistakes as they won't be overlooked once they've earned their doctorate.

Second-year medical students should always be ready to deal with whatever implications the board results may bring because of the barrier that stands tall when shifting from the basic years to the clinical years. If any difficulties should develop during the results, the batch members must band together and support one another.

Speaking of the initial days of clinical rotation, it feels like starting medical school all over again, but it's different because instead of just sitting in class and dissecting a dead body in dissection halls, students are engaged with actual patients in hospital settings. At first, the students are cautious, perplexed, and worried about the new environment and how to approach the patients but it's equally amazing when people start to recognize you as a doctor with your scrubs and white apron. The undergraduates genuinely realize the value of relationships and interactions with seniors after starting clinical postings because we hope they will share their knowledge with us as we strive to develop clinical skills. History-taking classes are conducted at the beginning of the clinical where students are taught to introduce themselves to the patient, obtain their consent, understand the method of collecting history and the significance of each of its components and approach patients with confidence rather than confusion. Through interactions with patients, they discover how to be humble, kind, empathic, and compassionate. They also discover that being a doctor requires not only having extensive knowledge but also being approachable.

After practising to take a history, the teachers in each department focus on teaching the general and clinical examinations in a step-by-step manner. As they learn the clinical examination of the relevant system and, after gaining enough confidence, perform the lessons at the patient's bedside, they experience a peak in their sense of progress toward becoming medical professionals. Instead of practising on real patients, they can perform the examination on their own friends and family while adhering to the guidelines in the standard books and references. After all, we can only successfully promote ourselves in this cutthroat market if we can turn all of our knowledge into a refined set of talents. The sense of the weight and responsibility of the profession inclines in the third year.

### 3. Extracurricular activities

The amount of reading, practising, and postings make students want to reconsider joining in medical school, but extracurricular activities bring this road to life, enhance self-esteem, and keep them motivated. Additionally, maintaining a healthy physical and mental lifestyle is crucial for medical students as aspiring physicians so that they may impart this information to others. Participation in extracurricular activities can also be linked to the emergence of selfcontrol systems that support favourable academic, psychological, and social results.^6^

MBBS is not just about studying; they have many possibilities to engage in a variety of extracurricular activities, such as sports activities, training, campaigns, advocacy efforts, health awareness projects, health camps, conferences, workshops, medical education programs, and mentoring juniors.^7^ By the third year, they are more familiar with these possibilities and understand the value of taking part in similar activities.

After obtaining a taste of both the basics and the clinical, they can focus on developing different areas of their CV, which would be clearly beneficial in this competitive environment.

## WAY FORWARD

To become a highly talented and promising professional, one must integrate clinical and basic scientific medical knowledge, practice at the patient's bedside, and hone communication skills. It is important for medical students to remember that they are the clinic's least experienced employees and are only there for educational purposes. They have to be careful not to misdiagnose or misprescribe a patient's condition. They should seize this chance and take full advantage of it in order to prevent regretting it later. Every medical student should believe that the only way to master anything is to practice it over and over until it comes naturally.
